# Development of a Microwave Sensor for Solid and Liquid Substances Based on Closed Loop Resonator

**DOI:** 10.3390/s21248506

**Published:** 2021-12-20

**Authors:** Aiswarya S, Sreedevi K. Menon, Massimo Donelli, Meenu L

**Affiliations:** 1 Center for Wireless Networks & Applications (WNA), Amrita Vishwa Vidyapeetham, Amritapuri 690525, India; aiswaryas@am.amrita.edu (A.S.); meenul@am.amrita.edu (M.L.); 2 Department of Electronics and Communication Engineering, Amrita Vishwa Vidyapeetham, Amritapuri 690525, India; 3 Department of Civil Environmental and Mechanical Engineering, University of Trento, 38123 Trento, Italy; massimo.donelli@unitn.it

**Keywords:** adulteration, dielectric permittivity, filter, resonator, sensitivity, sensor

## Abstract

In this work, a compact dielectric sensor for the detection of adulteration in solid and liquid samples using planar resonators is presented. Six types of filter prototypes operating at 2.4 GHz are presented, optimized, numerically assessed, fabricated and experimentally validated. The obtained experimental results provided an error less than 6% with respect to the simulated results. Moreover, a size reduction of about 69% was achieved for the band stop filter and a 75% reduction for band pass filter compared to standard sensors realized using open/short circuited stub microstrip lines. From the designed filters, the miniaturised filter with Q of 95 at 2.4 GHz and size of 35 mm × 35 mm is formulated as a sensor and is validated theoretically and experimentally. The designed sensor shows better sensitivity, and it depends upon the dielectric property of the sample to be tested. Simulation and experimental validation of the designed sensor is carried out by loading different samples onto the sensor. The adulteration detection of various food samples using the designed sensor is experimentally validated and shows excellent sensing on adding adulterants to the original sample. The sensitivity of the sensor is analyzed by studying the variations in resonant frequency, scattering parameters, phase and Q factor with variation in the dielectric property of the sample loaded onto the sensor.

## 1. Introduction

In the last decades, there has been a growing interest in microwave sensors [1] in various domains of science and engineering. The evolution of monolithic microwave integrated circuits (MMIC) able to operate at very high frequency bands make possible the design of microwave systems and components with high performances while at the same time keeping the cost at a reasonable level. In particular, microwave sensors [2] are of great interest because they are able to detect different physical environmental parameters with a high degree of accuracy [3] at the cost of RFIDs tags [4,5,6]. Thus, microwave sensors have found diverse applications in various fields such as healthcare monitoring [7,8], wearable applications [9], gas sensors [10], temperature sensing [11,12], humidity sensing [13] and adulteration detection [14]. The versatility and reliability of these sensors makes it applicable in these diverse areas.

The paper focuses on microwave sensors based on the frequency domain because they are low cost, easy to fabricate and more flexible. In particular, more focus is given to microstrip technology to develop compact planar resonators [15] with reliable performance for sensor applications. Different microwave devices such as antennas, filters, couplers and amplifiers can be designed using planar transmission lines [16,17,18]. The paper discusses a set of planar resonators which can act as stop or pass band filters. The advantage is that the same design techniques can be considered for the filters as well as for sensors. Various steps are discussed in the paper which aim to reduce the physical dimension of the filters which acts as sensors. The goal is to obtain compact devices characterized to have good capabilities to detect variations in the dielectric permittivity of substances placed on the surface of the device.

Filters are important components of any telecommunication systems, and they are also fundamental for microwave sensors. Filter design can be easily accomplished using microstrip lines [19] or coplanar wave guides [20,21,22], which are the most commonly used planar transmission lines. Microstrip line (MSL) assisted filters are much preferred due to the ease of design and realization. An absorptive band stop filter can be realized using two quadruple couplers and band pass filters [23]. The circuit implementation of narrow band pass, elliptic and microstrip filters was presented in [24] and can be used to realize band pass filters at 2 GHz [25]. The design of an open-loop resonator based filter antenna for Wi-Fi applications is presented in [26,27]. The bandwidth (BW) of a filter is influenced by the design of the resonators, which can provide ultra-wide rejection bands [28]. High-performance miniaturized structures are preferred to Monolithic Microwave Integrated Circuits (MMICs), where a coplanar wave guide (CPW) is preferred. The performance of coplanar-based microstrip patches was reviewed in [29]. The design of a filter-antenna, composed by the integration between a filter and an antenna, is discussed in [30], which constrains the antenna’s operation only in the desired frequency range. Passive filters work on the principle of back scattering or resonance. An approximation of the equivalent circuit of the filters was shown in [31]. Reconfigurable filters can work in different frequency ranges, with good tunability as in [32]. Dual-band operation facilitates a circuit to work efficiently at two different frequencies. Hairpin resonators were found to provide dual-band characteristics [33]. Variations in the geometry of the system gives affordable band-pass and band-reject characteristics [34]. The applications of filters for the design of integrated circuits were described in [35,36]. This paper discusses the design and miniaturization of planar filters for sensor applications.

Radio Frequency (RF)-based planar sensors finds diverse applications due to their reusability and repeatablity. A review about microwave planar-resonator-based sensors in [37] highlights the various advantages and applications of microwave sensors. The highly sensitive RF-based planar resonators can be used for the accurate measurement of the complex permittivity of material samples. The dielectric measurements of solids and liquids were described in the paper. There is even a possibility of non-invasive glucose monitoring using planar RF sensor [38]. The works in [39,40,41] give a wider view on non-invasive glucose monitoring. Research is ongoing for the development of non-invasive continuous blood glucose monitoring sensors [39]. A split-ring resonator is used in the sensing part for measurements in their sensor. They conducted studies in real time on non-diabetic individuals, and only a narrow range of blood glucose levels is studied. Even the glucose monitoring by sensing the characteristics of human tongue studies are going on [40]. These studies are to be conducted in a wide range of individuals for the accuracy and efficiency calculation. Ref. [41] presents a four-cell CSRR hexagonal configuration for measuring the glucose level, and the sensitivity of the design is also included.

Another application of the microwave sensor is the characterization of concrete samples of building materials and is discussed in [42]. The design uses a planar antenna operating at multiple bands, and the resonance at 2.25 GHz is used for the sensor application. The variation of operating frequency for different dielectric permittivity is the key component in the study. The work in [43] discusses a microwave planar sensor for the permittivity determination of the dielectric materials at a 4 GHz operating frequency. Designed on Rogers RT Duriod, accuracy analysis of the sensor is also studied in the paper. A similar work to the above is discussed in [44], in which a Sierpinski fractal-curve-based structure is design, fabricated and validated. The variation of frequency for different dielectric permittivity values is being studied and experimentally validated in the discussed paper. A simple microwave resonator array integrated with liquid metal is discussed in [45], which is used for the accurate dielectric sensing using a modified split ring resonator. This designed sensor offers a better sensitivity of 30 MHz, with less than a 3 dB variation in the resonant amplitude with the advantage of tunability. The work in [46] also discusses the multi-band RF planar sensor fabricated on FR4 epoxy operating in microwave sensors operating at different bands, namely, 1.5 GHz, 2.45 GHz, 3.8 GHz and 5.8 GHz. With the extensive literature survey, the application of planar-resonator-assisted sensors for detecting adulteration in food is carried out in this paper.

The major problem that the common people are facing is their health issues. One of the major reason for the health issues is due to the intake of adulterated foods. It would be very beneficial if there was a system to detect the adulteration in food. An emerging research area is the design and use of planar radio frequency designs which can act as sensors. An antenna can be used in testing adulteration [47], in which an OLR-loaded self complementary dipole antenna operating in the band 1.1 GHz to 3.3 GHz is used as the sensor. It is used as a liquid sensor, in which the dilution of milk with water, adulteration of coconut oil with rice bran oil and adulteration of honey with sugar syrup is tested using the sensing antenna. Similarly, other antennas, resonators, filters, etc., can be used as a sensor if it shows good sensitivity on loading the samples on to sensor.

Sensitivity is the major property of a sensor which has to addressed while designing and testing. In general, sensitivity of a sensor can be defined as the output changes of a sensor when the input quantity is being changed. When considering RF planar sensors, the sensitivity parameter may depend upon one or more parameters. From literature, we can find sensors based on resonant frequency, insertion/return loss, phase or quality factor (Q) [48]. The resonant frequency is an important parameter that has a direct relation with the sensitivity of the resonator structures [49,50,51]. The sensing principle is based on the measurement of insertion loss and is using complementary split ring resonators (OCSRRs) for the dielectric characterization and measurement of solute concentration in liquid [52]. Microwave-based microfluidic sensors discussed in [53] for glucose monitoring in aqueous solution is initiated from a band stop filter and is a biosensor consisting of a quarter wave-length stub implemented in a thin film microstrip technology. The measurement of glucose concentration using the Q factor of a sensor is presented in [54]. Similarly, the resonator in [55] has used Q factor for dielectric characterization in metamaterials. The phase of a resonating structure also has an effect on the permittivity and material characteristics [56,57].

This paper discusses the design fabrication and assessment of compact microwave filter designs and analyzed the perfect design which can operate as a microwave sensor in the frequency domain. The proposed filter designs were first numerically assessed with a commercial software based on finite element methods FEM (namely, ANSYS HFSS) and then experimentally assessed in controlled environments. The sensor validation of the compact designs is carried out using simulation software ANSYS HFSS, and the best filter which can operate as sensor is experimentally tested and analyzed. In the experimental assessment, a sensor able to detect the variations in dielectric permittivity has been proposed and analyzed. The sensor is based on a planar resonator, printed on a dielectric substrate, which is able to change its resonance frequency versus the dielectric permittivity. The dielectric permittivity variation is obtained by loading samples onto the surface of the resonator. This concludes that the designed sensor is a dielectric permittivity sensor made with microwave techniques. To experimentally assess the presented sensor a measurement campaign has been carried out considering different substances and mixture. The obtained results are quite promising, and they demonstrate the capabilities and the potentialities of the proposed sensor to detect adulteration in different substances. All these design procedures are explained in detail in the paper. The work is structured as follows: Section 2 is devoted to the design and miniaturization of microwave sensors based on closed loop resonators. The designed sensors are also numerically and experimentally assessed in Section 2.1 and Section 2.2, respectively. The simulation analysis of the miniaturized resonators as microwave sensors is explained in Section 3. The sensing capabilities of sensors with the best performances are experimentally assessed by loading different substances in controlled environment in Section 4. Experimental testing and results using the proposed sensor in solids and liquids and adulteration sensing in food components is presented in Section 4.1 and Section 4.2, respectively, and the analysis of experimental results and comparisons results are discussed in Section 4.3. Finally, Section 5 reports the conclusions and ideas for future works.

## 2. Sensor Design and Miniaturization

This section addresses the design and miniaturization of microwave sensors, starting from resonators providing band pass and band stop filter responses. Without loss of generalization, a low frequency band, which presents a high problem concerning the miniaturization in microstrip technology, has been considered. In particular, the sensor design begin as a standard second-order butter worth band pass/stop filter [58,59,60], in particular the following characteristics: central frequency $f_{w}=2.48$ GHz, bandwidth $B_{w}=1.5$ GHz and $Z_{in}=Z_{out}=50$ Ω have been considered. The design frequency is chosen as 2.48 GHz, as it is a low frequency which leads to a large wavelength and permits demonstrating that a very compact device can be obtained despite the low frequency used. The design of these devices is quite trivial, and it follows the classical and well-known insertion loss method described in [61] and given by the following relation:

(1) $$IL\left(\omega \right)=10log\frac{P_{in}\left(\omega \right)}{P_{out}\left(\omega \right)}=-10log\left(1-{\left|\Gamma (\omega )\right|}^{2}\right)=-20log\left|S_{21}\left(\omega \right)\right|$$

**Figure 1:**
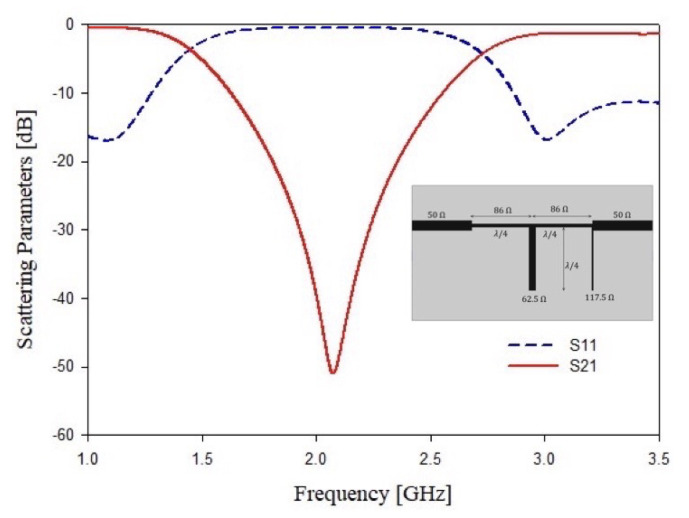
Second order standard stop band filter. Microstrip implementation and simulated scattering parameters $S_{11}$ and $S_{21}$.

**Figure 2:**
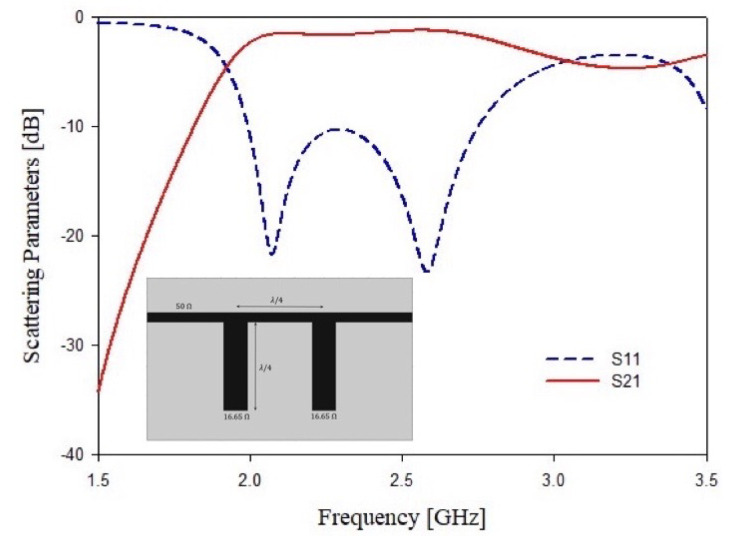
Second order standard band pass filter. Microstrip implementation and simulated scattering parameters $S_{11}$ and $S_{21}$.

As stated above, starting with the standard design of a band stop and a band pass filters both operating at 2.48 GHz with a bandwidth of $B_{w}=1.5$ GHz and an attenuation of A=−30 dB at the central frequency and a ports impedance of $Z_{in}=Z_{out}=50$ Ω. A filter order N=2 is enough to reach the requirements in terms of scattering parameters. The second step of the design consists of the transformation from lumped elements into microstrip technology. This goal can be achieved by removing all the series’ reactive elements with shunt stubs by applying the Kuroda’s and Richard’s transformations in order to obtain a commensurate microstrip stubs filter [61]. The microstrip filters have been implemented considering a commercial FR4 dielectric substrate of thickness t=1.6 mm, tan(δ)=0.02 and $\varepsilon_{r}=4.0$. The obtained microstrip filter layouts and their simulated responses are reported in Figure 1 and Figure 2, respectively. The main problem of these structures is the total length and the stubs’ dimension. In particular, considering the permittivity of dielectric substrate and the low central frequency band, the stop band filter shows a total length of $\frac{\lambda }{2}$, which corresponds to about L=90.7 mm of total filter length and W=45.1 mm of width, while the band pass filter has a length of the filter of L=90.75 mm and a width of W=60.25 mm. These dimensions are too high, especially if different resonators must be enclosed in a single design. Therefore, a miniaturization is mandatory. In the next sub-section, different structures will be considered to obtain a good resonator miniaturization.

### 2.1. Resonators Design and Miniaturization

**Figure 3:**
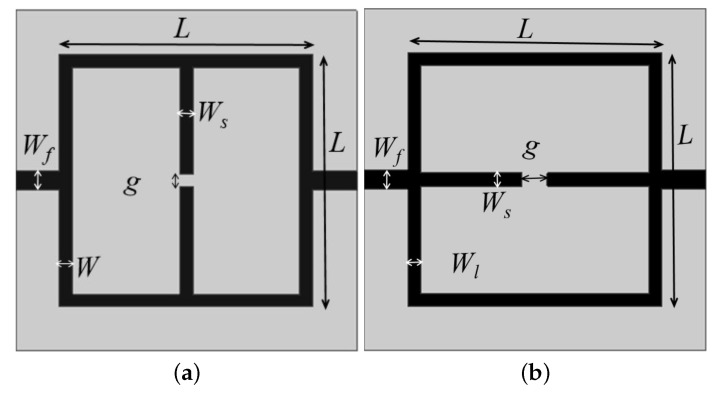
Geometry of the first two sensors realized with closed loop resonators and stubs loading: (**a**) perpendicular; (**b**) parallel to microstrip feeding line.

The second order band pass and band stop filters designs are optimized and miniaturized to a closed loop structure, but the structure fails to operate as a resonator. So, parallel and perpendicular stubs are added on to the closed loop resonator to make it operational as a resonator. In this section, different resonator geometries with stop and band pass behavior are designed in microstrip technology and numerically assessed. The first two considered geometries are reported in Figure 3, where a closed-loop resonator with a perpendicular (a) and parallel (b) to the signal line open end stubs are shown. The introduction of the stubs permits a good miniaturization. The geometries reported in Figure 3 are quite similar, and they present four different geometrical parameters: the side length *L* and the width *W* of the main square resonator and the gap *g* and the thickness $W_{s}$ of the two open end stubs.

**Figure 4:**
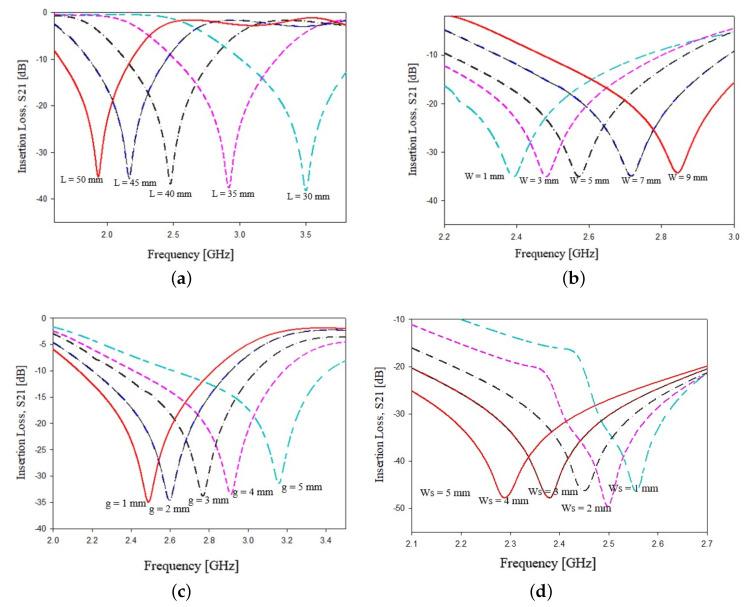
Dependence of the main frequency peak versus variations of geometrical parameters: (**a**) length of the main square loop side *L*, (**b**) thickness of the square loop microstrip *W*, (**c**) gap between the two stubs *g*, (**d**) thickness of the stubs microstrip $W_{s}$.

The parametric study of each of the design parameters of the resonators is carried out for different values of dielectric constants, and based on these calculations, the formulas for design equations are derived theoretically, which is explained in detail below. The following formula can be used to estimate the central frequency of the structures reported in Figure 3 which behave as band stop filters:

(2) $$f_{w}=\frac{\gamma_{1}Cg}{2LW_{s}\sqrt{\frac{\varepsilon_{r}+1}{2}+\frac{\varepsilon_{r}-1}{2\sqrt{1+\frac{12h}{w}}}}}$$

where *C* is the light velocity in vacuum, $\varepsilon_{r}$ and *h* are the relative dielectric permittivity and the thickness of considered substrate, and $\gamma_{1}$ is an empirical weighting factor which depends on the substrate dielectric permittivity. In particular, $\gamma_{1}=2.28$ for $3.2\leq \varepsilon_{r}<4.0$, $\gamma_{1}=2.25$ for $4.0\leq \varepsilon_{r}<6.0$, $\gamma_{1}=2.30$ for $6.0\leq \varepsilon_{r}<9.2$ and $\gamma_{1}=2.20$ for $9.2\leq \varepsilon_{r}<10.2$. *L* is the length of the side of the square loop, *g* is the gap between stubs and *w* and $W_{s}$ are the microstrip thicknesses of the square resonator and stubs, respectively. To assess the influence of the above geometrical parameters, a parametric analysis has been carried on. In particular, without loss of generality, the two geometries reported in Figure 3 have been designed, following Equation (2) to resonate at $f_{w}=2.48$ GHz and simulated with a commercial software, namely, ANSY HFSS. The considered dielectric substrate was FR4 ($\varepsilon_{r}=4.0$, tan(δ)=0.02, h=1.6 mm). The geometrical parameters $L,W,W_{s}$ and *g* have been varied in a given range to observe the variations of the main resonance frequency peak. From the data reported in Figure 4a, which reports the frequency peak variations versus the length side *L* of the main square resonator, it can be observed as expected that a reduction in the side length *L* corresponds to higher values of the frequency peak. A similar behavior is observed in Figure 4b when the width *W* of the square resonator is increased, in particular an increment of about 200 MHz can be observed for increments of 2 mm. Concerning the stubs gap *g*, an increment of 1 millimeter leads to a frequency shift of about 100 MHz, as reported in Figure 4c. The increment in the stubs width $W_{s}$ has an inverse effect on the frequency shift. In particular, an increment of 1 millimeter on $W_{s}$ leads to a reduction in the frequency peak of about 150 MHz as reported in Figure 4d. It is worth noticing that the data reported in Figure 4 refer to the geometry of Figure 3a, however, very similar results have been obtained for the geometry of Figure 3b.

**Figure 5:**
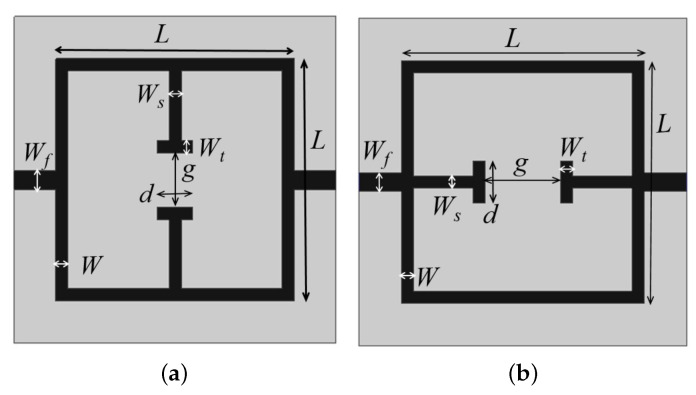
Geometry of the other two sensors realized introducing a T-shaped on the stubs head: (**a**) perpendicular, (**b**) parallel to microstrip feeding line.

To further increase the level of miniaturization, a T-shaped stubs modification has been considered. In particular, two segments of microstrip with a width $W_{t}$ were placed perpendicular to the stub main line as reported in Figure 5a,b. The introduction of the T-shape should introduce two new degrees of freedom to the geometrical parameters, the length *T* and the width $W_{t}$, as indicated in Figure 5. The introduction of two new geometrical parameters leads to the following formula, which is the design equation derived by analyzing the parametric variation of different values of dielectric permittivity and is aimed to estimate the resonance peak:

(3) $$f_{w}=\frac{\gamma_{2}CgW\sqrt{\frac{\varepsilon_{r}+1}{2}+\frac{\varepsilon_{r}-1}{2\sqrt{1+\frac{12h}{w}}}}}{2LW_{d}}$$

where $\gamma_{2}$, similarly to relation (2), is an empirical weighting factor which depends on the substrate dielectric permittivity. In particular, $\gamma_{2}=1.50$ for $3.2\leq \varepsilon_{r}<4.0$, $\gamma_{2}=1.14$ for $4.0\leq \varepsilon_{r}<6.0$, $\gamma_{2}=0.95$ for $6.0\leq \varepsilon_{r}<9.2$ and $\gamma_{2}=0.39$ for $9.2\leq \varepsilon_{r}<10.2$.

**Figure 6:**
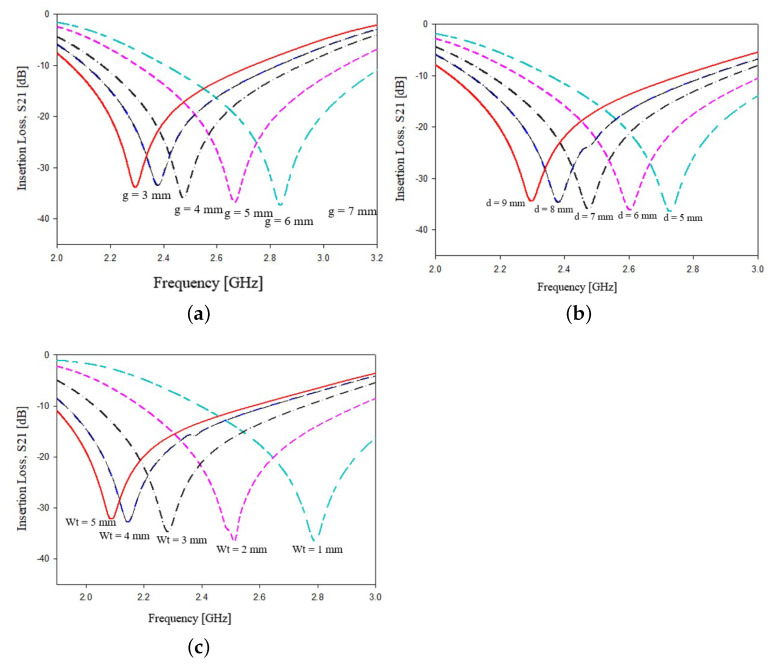
Dependence of the main frequency peak versus variations of geometrical parameters, with reference to Figure 5: (**a**) gap between the stubs *g*, (**b**) length of stubs tips *d* and (**c**) width of the stubs tips $W_{t}$.

The effects of geometrical parameters L,W and $W_{s}$ versus resonance frequency, for the prototypes reported in Figure 5a,b are very similar to those obtained for geometries reported in Figure 3a,b therefore only the additional parameters g,d and $W_{t}$ were analyzed to evaluate their effects on the resonance frequency. The results of the parametric analysis is reported in Figure 6a–c. The data reported in Figure 6a–c show the frequency peak variations versus the stub gaps *g* (a), stub tip lengths *d* (b) and stub tip widths $W_{t}$ (c), respectively. In Figure 6a, it is observed that when the stubs gap *g* is increased by 1 mm, an increment of about 100 MHz in the frequency peak can be observed. Concerning the other two parameters, an increment of 1 millimeter of the stub tip lengths *g* and of the stub tip widths lead to a frequency shift reduction of about 50 MHz, as shown in Figure 6bc, respectively. As in the previous cases, very similar results have been obtained for the other geometrical parameters of configurations reported in Figure 5a,b.

**Figure 7:**
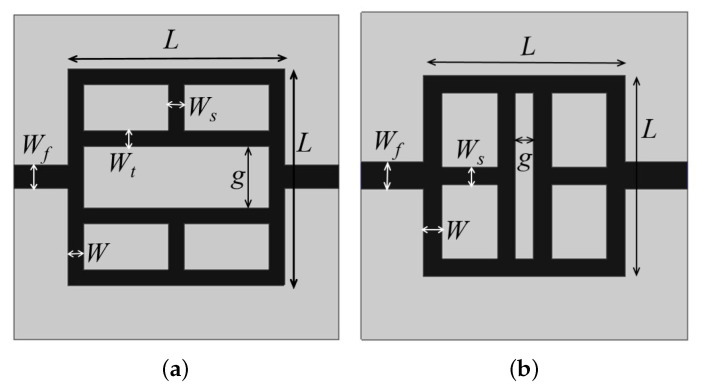
Geometry of the last two sensors realized with closed loop resonators and stubs tips of the same square resonator side length: (**a**) perpendicular (**b**) parallel to microstrip feeding line.

**Figure 8:**
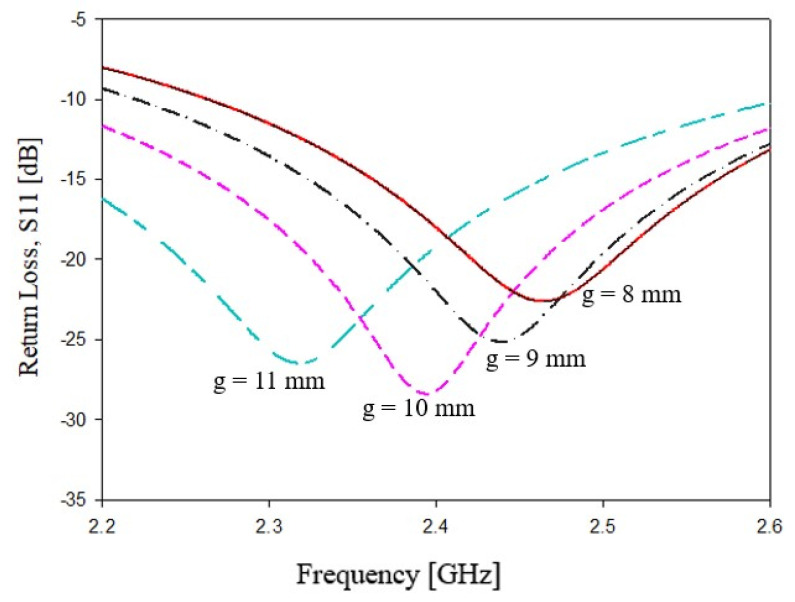
Dependence of the main frequency peak versus variations in stubs gap *g*, with reference to Figure 7.

The last two considered configurations are reported in Figure 7a,b. The T-shaped stubs, parallel and perpendicular to the feed line, were modified to obtain further size reductions. In these configurations, the width of the stub tips *d* is equal to the square side *L*. In addition, in this case of the empirical prediction formula, which is the design equation derived by analyzing the parametric variation on different values of dielectric permittivity, is reported in the following:

(4) $$f_{w}=\frac{\gamma_{3}C}{2gLW_{t}\sqrt{\frac{\varepsilon_{r}+1}{2}+\frac{\varepsilon_{r}-1}{2\sqrt{1+\frac{12h}{w}}}}}$$

where $\gamma_{3}$, similarly to relations (2) and (3), is an empirical weighting factor which depends on the substrate’s dielectric permittivity $\varepsilon_{r}$. In particular, $\gamma_{3}=0.600$ for $3.2\leq \varepsilon_{r}<4.5$, $\gamma_{3}=0.705$ for $4.5\leq \varepsilon_{r}<6.0$, $\gamma_{3}=0.601$ for $6.0\leq \varepsilon_{r}<9.2$ and $\gamma_{3}=0.602$ for $9.2\leq \varepsilon_{r}<10.2$. In these configurations, we have a reduction in the degrees of freedom. The only geometrical parameter that requires a parametric analysis is the stub gaps *g*. The results of the parametric analysis for *g* are reported in Figure 8. As it can be observed, a stubs distance *g* increment of about 1 mm leads to a reduction in the resonance peak of about 50 MHz.

**Figure 9:**
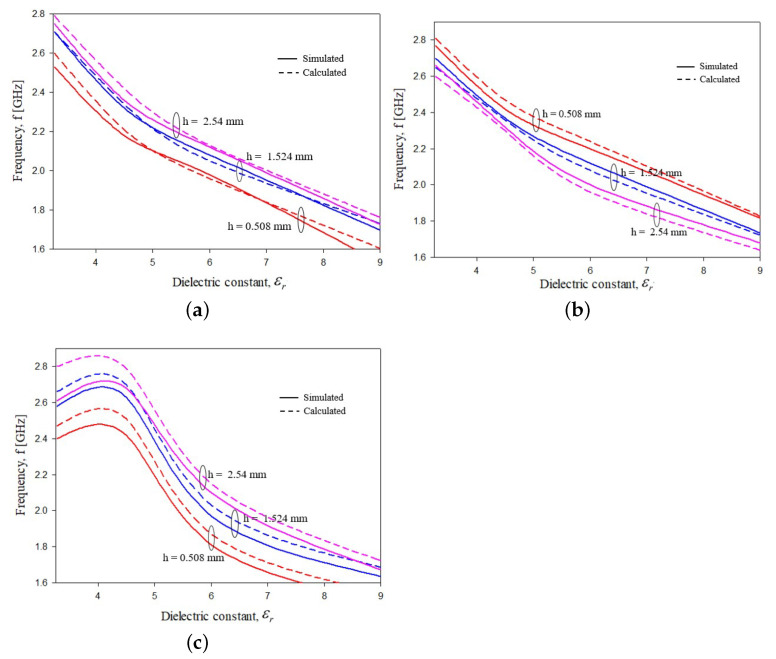
Validation of the empirical relations (2)–(4). Main resonance peak vs. substrate dielectric permittivity $\varepsilon_{r}$ comparisons between semi-analytical formulas and numerical simulations, (**a**) configuration in Figure 3, (**b**) configuration in Figure 5 and (**c**) configuration in Figure 7.

To validate relations (2)–(4), a numerical simulation campaign has been performed. In particular, the resonance peak of each the six considered configurations has been estimated for different values of substrate permittivity. In particular, $\varepsilon_{r}$ was varied in the range of $3.0\leq \varepsilon_{r}\leq 9.0$[62] to cover almost all possible commercial dielectric substrates. The values obtained with the semi-analytical relations (2)–(4) were compared with the numerical data obtained with a commercial software, namely, HFSS. The results are reported in Figure 9a, Figure 9b and Figure 9c, respectively. As it can be noticed, the agreement between simulated, numerical and predicted semi-analytic values is quite satisfactory in the whole considered dielectric permittivity range and for all geometrical configurations. The overall error between numerical and semi-analytical values was always below 5%.

After the miniaturization phase, where the geometrical dimensions and the devices characteristics have been accurately tuned, the final geometrical configurations have been obtained and summarized in Table 1. It is worth noticed that prototypes 1, 2, 3 and 4 show a band pass filter behavior, while prototypes 5 and 6 act as a stop band filter. In particular, the parameters *L*, *g* and *d*, whose dimensions are reported in mm, are compared. Concerning the other geometrical parameters, they were fixed to W=3.9 mm, $W_{f}=3.0$ mm and $W_{t}=2.0$ mm. A dielectric substrate ($\varepsilon_{r}=4.0,tan\left(\delta \right)=0.02,h=1.6$ mm) has been considered for all the configurations.

A comparison of the scattering parameters, bandwidth, total length and covered area are reported in Table 2 and Table 3, respectively. In particular, Table 2 compares prototypes with a stop band behavior, while Table 3 compares the prototypes with a pass band response. As it can be noticed from the data reported in Table 2 and Table 3, the best design for the band stop and band pass are prototypes 4 (F4) and 6 (F6), respectively. In particular, device 4 presents the maximum band rejection with reduced prototype size of 1225 mm${}^{2}$. Similarly, device 6 acts as the best band pass filter with a wide bandwidth of BW=1.6 GHz and a reduced area of A=1303 mm${}^{2}$. For the sake of comparison in Table 4, different state-of-the-art filters and sensors, reported in the scientific literature, are compared together. The data reported in Table 4 demonstrate that the performance of prototypes 4 and 6 outperforms state of the art devices in terms of bandwidth and compactness. The provided filter designs F4 and F6 lead to reduced size, with respect to the ones already presented in scientific literature, in particular, we obtained a size reduction of about 69% was achieved for the band stop filter and 75% reduction for band pass filter design. Thus, the compactness in design is achieved compared to the initial designs discussed in the paper, as well as from the discussed literature.

### 2.2. Experimental Assessment

**Figure 10:**
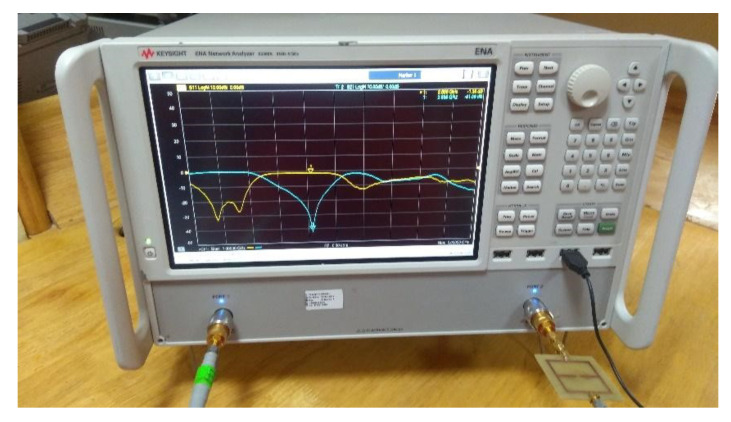
Photo of the considered experimental setup.

**Figure 11:**
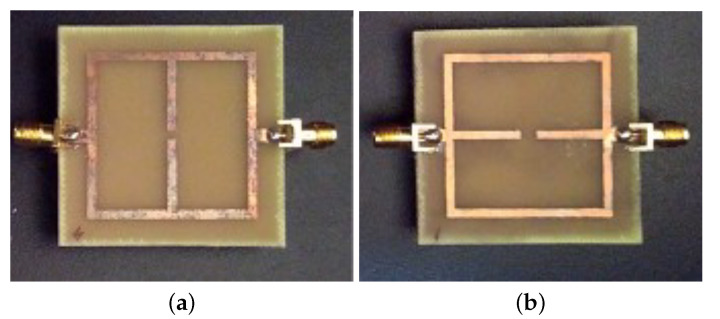
Photo of the first two prototypes reported in Figure 3 equipped with two SMA coaxial connectors: (**a**) stubs perpendicular and (**b**) parallel to the microstrip feeding line.

**Figure 12:**
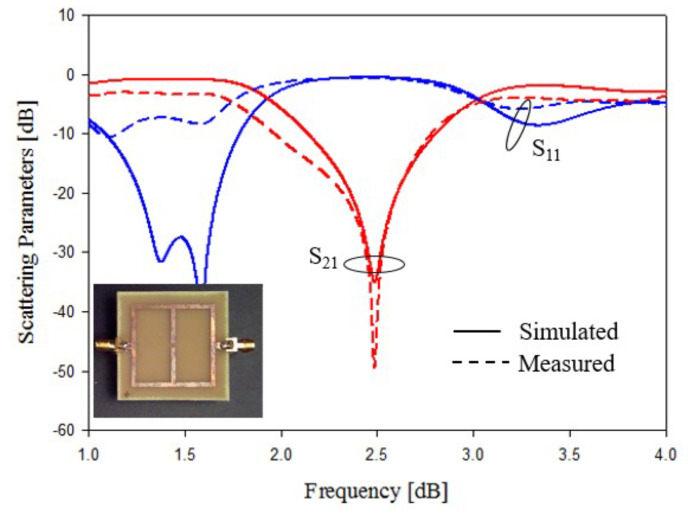
Experimental assessment, prototype of Figure 11a. Comparisons between numerical and measured scattering parameters.

**Figure 13:**
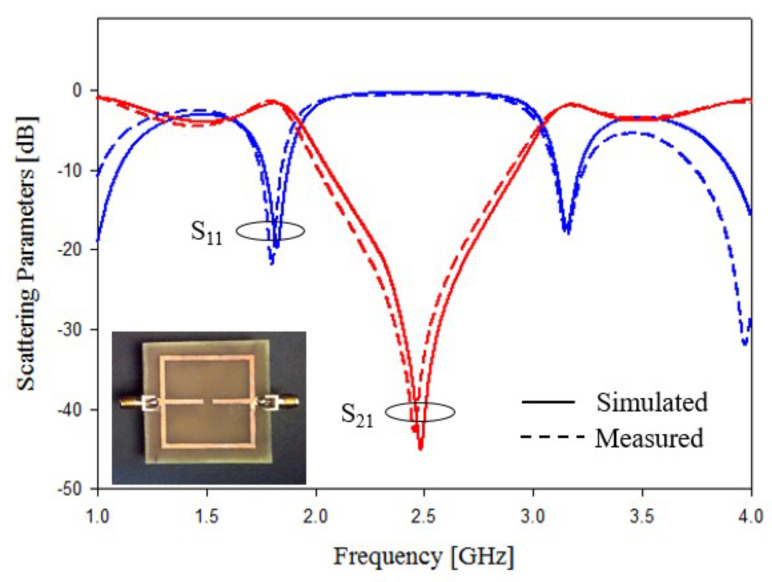
Experimental assessment, prototype of Figure 11b. Comparisons between numerical and measured scattering parameters.

In this subsection, an experimental assessment has been carried out. The six prototypes developed in the previous sections have been fabricated with a photolithographic process, a FR4 commercial dielectric substrate ($\varepsilon_{r}=4.0$, tan(δ)=0.02, h=1.6 mm ) has been considered. Concerning the experimental setup, the scattering parameters of the sensors were experimentally measured with a Vector Network Analyzer, namely, a Keysight E5080A, and the prototypes have been equipped with two subminiature type A connectors (SMA). A photo of the considered experimental setup is reported in Figure 10, while the photos of prototypes shown in Figure 3a,b are reported in Figure 11a,b, respectively. The scattering parameters, the return loss $S_{11}$ and the insertion loss $S_{21}$ have been measured with the vector network analyzer and compared with numerical results obtained with the HFSS software. Figure 12 and Figure 13 report the comparisons between the numerical and experimental results for the prototypes with stubs perpendicular and parallel to the feeding line, respectively.

**Figure 14:**
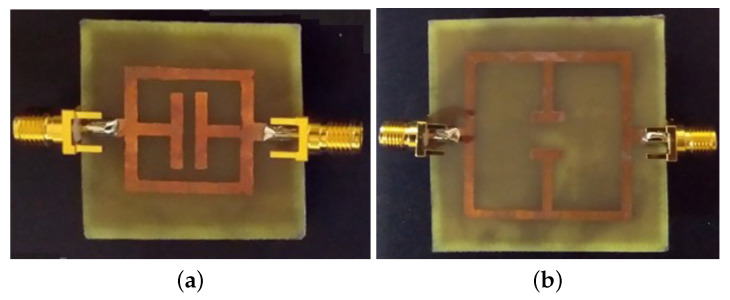
Photos of the prototypes with T-shaped stubs (**a**) parallel and (**b**) perpendicular to the microstrip feeding line.

As it can be noticed from the data of Figure 12 and Figure 13, the agreement between numerical and experimental results is quite good. The main frequency peak position and the bandwidth requirement satisfy the requirements. In the following experiment, prototypes with T-shaped stubs, parallel and perpendicular to the feeding line, whose reference geometries are reported in Figure 5a,b are experimentally assessed. The prototypes photos are reported in Figure 14a,b, respectively.

**Figure 15:**
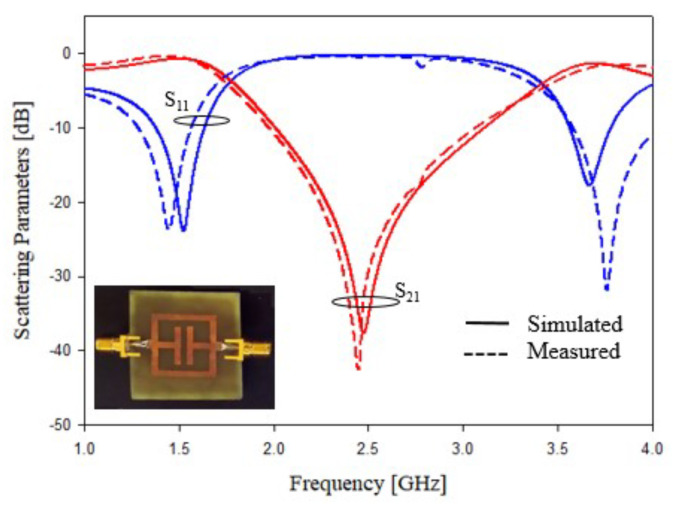
Experimental assessment, T-shapes stubs, prototype of Figure 5a. Comparisons between numerical and measured scattering parameters.

**Figure 16:**
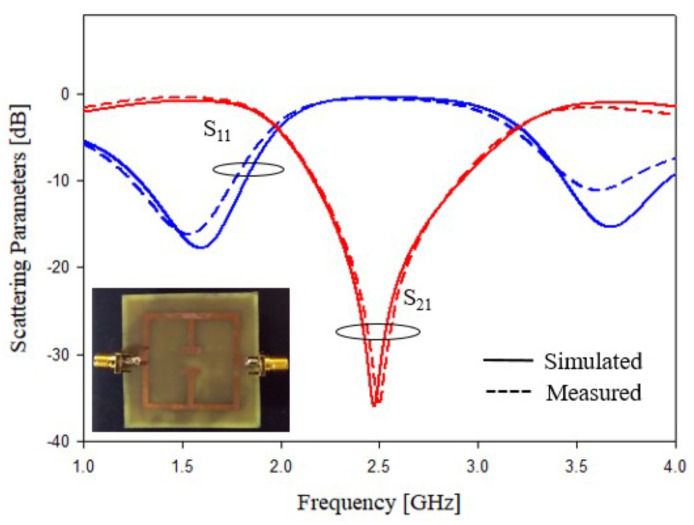
Experimental assessment, T-shapes stubs, prototype of Figure 5b. Comparisons between numerical and measured scattering parameters.

The experimental assessment of prototypes reported in Figure 5a,b are shown in Figure 15 and Figure 16. In addition, in this case, the agreement between numerical and experimental results is quite satisfactory. The obtained results demonstrated the effectiveness of empirical design relations (2) and (3). Only a slight frequency shifts can be observed in the data reported in Figure 16, and the frequency shifts are probably due to the dielectric material tolerances.

**Figure 17:**
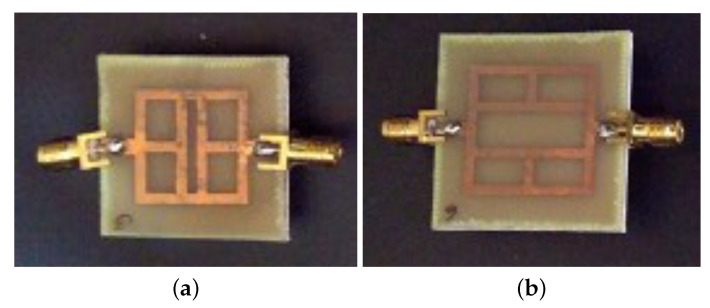
Photos of the prototypes with T-shaped stubs: (**a**) T-stubs length equal to *L* perpendicular and (**b**) parallel to the microstrip feeding line.

**Figure 18:**
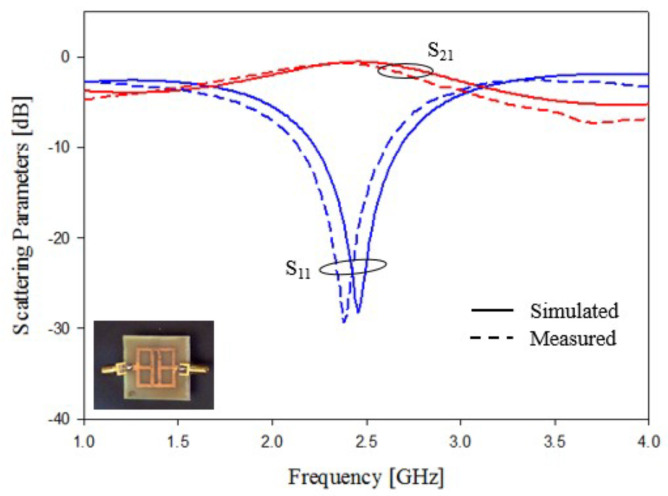
Experimental assessment, T-shaped stubs of length equal to *L* perpendicular to the feeding line, prototype of Figure 7a. Comparisons between numerical and measured scattering parameters.

**Figure 19:**
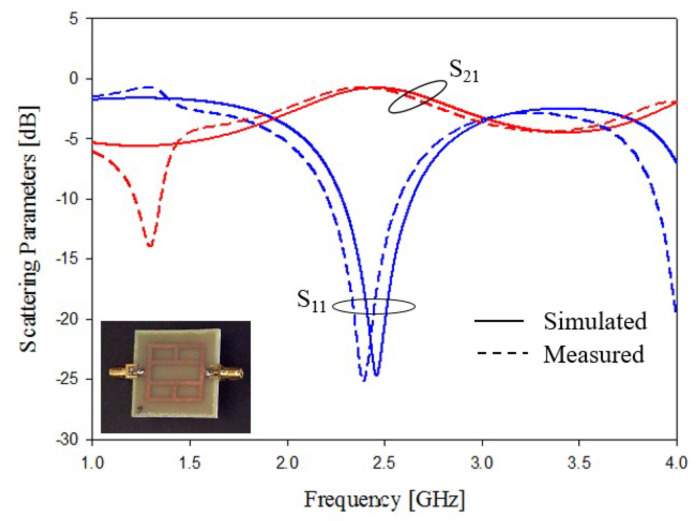
Experimental assessment, T-shaped stubs of length equal to *L* perpendicular to the feeding line, prototype of Figure 7b. Comparisons between numerical and measured scattering parameters.

In the last experimental assessment, the prototypes characterized with a T width equal to the length of the square side of the resonator are characterized in terms of scattering parameters. The prototype photos related to the geometries shown in Figure 7a,b are reported in Figure 17a,b. In addition, for the last two prototypes, the scattering parameters have been measured and compared with the numerical results obtained with the HFSS software. The scattering data are reported in Figure 18 for geometry of Figure 5a and Figure 19 for the prototype of Figure 5b. The data reported in Figure 18 and Figure 19 show a frequency shift between the numerical and experimental of about 50 MHz, however, the agreement is quite satisfactory, and it demonstrates the correctness of the considered design procedure. For the sake of comparison, the performances of prototypes of Figure 5b and Figure 14a are compared in Table 5. The above prototypes show the best characteristics in terms of compactness and bandwidth. In particular, calculated, simulated, measured and predicted with the empirical design equations, the frequency peaks are reported in Table 5. As it can be noticed from the comparison reported in Table 5, the agreement is very good.

## 3. Simulation Analysis of Parallel Stub (PS) Filter as Microwave Sensor

**Figure 20:**
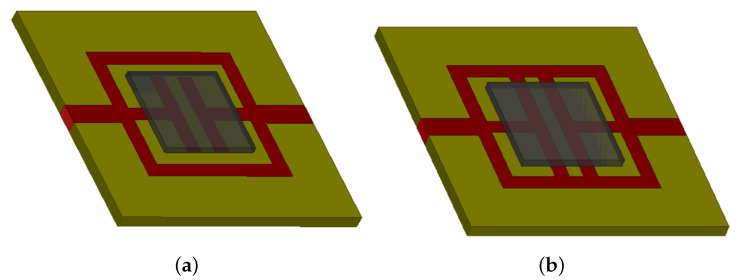
Schema of the two considered sensors with T-shaped stubs: (**a**) parallel stub (PS) and (**b**) parallel stub loop (PSL).

**Figure 21:**
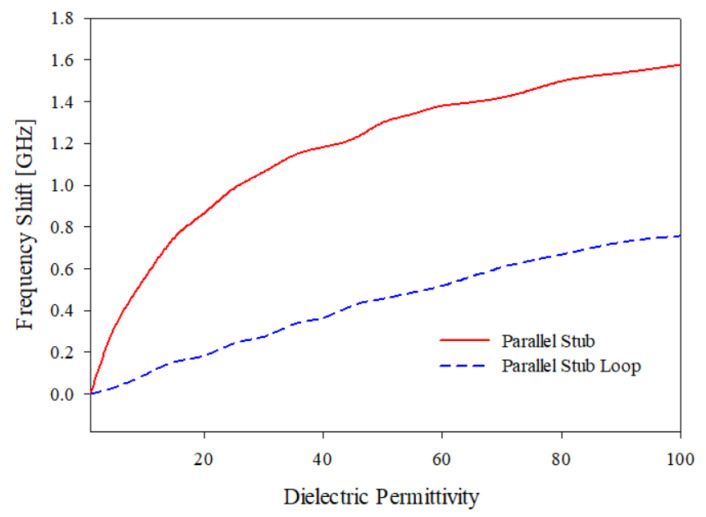
Sensing applications, T-shaped stubs and T-shaped stub of length equal to *L* parallel to the feeding line. Frequency shift versus dielectric permittivity of liquid substances placed on the resonator top side.

In this section, the sensing capabilities of the optimized prototypes developed in the previous section are investigated. In particular, the parallel T-shaped stubs F4, shown in Figure 5b which operate as band stop filters, and the parallel stubs loop F6, shown in Figure 7b which operate as band pass filters ($\varepsilon_{r}=4.0$, tan(δ)=0.02, h=1.6 mm), are considered because they show good performances and a very compact size. The prototypes can be used to estimate the dielectric permittivity of liquid or solid samples placed on the top side of the square resonators as reported in Figure 20a,b. The black areas in Figure 20, represent the areas where liquid or solid samples can be placed. In the fabricated prototypes, black shaded areas are covered with a small thickness of insulating glue, which do not significantly change the frequency peak. The introduction of a given substance on the top of square resonators will produce a frequency shift that can be used to estimate the of dielectric permittivity $\varepsilon_{rs}$ of the sample itself. An interesting application of such sensors is the detection of milk adulteration. A sample material having the same characteristic of the adulterated milk can be created following the indications reported in [63]. To estimate the sensors’ capabilities, a set of simulations with HFSS has been performed, and the results are reported in Table 6 and in graphical form in Figure 21. The results reported in Figure 21 show the frequency shift with the dielectric permittivity $\varepsilon_{rs}$, the PS sensor shows a very good linearity with respect to the dielectric permittivity $\varepsilon_{rs}$ variations, and it is the best candidate as a microwave sensor to detect the adulteration of different substances as it acts as a parallel plate capacitor. The equation of the capacitance of a parallel plate is given by:

(5) $$C=\frac{\varepsilon_{0}\varepsilon_{r}A}{d}$$

where $\varepsilon_{r}$ is the dielectric constant of the material, $\varepsilon_{0}$ is the permittivity of the free space, *A* is the area of the plate and *d* is the separation between the two plates [64]. As per the equation, when a new material is loaded between the stubs, the dielectric permittivity becomes varied, thus leading to a shift in capacitance and the resonant characteristics of the parallel stub providing high sensitivity to the gap between the parallel stubs where the samples are loaded. So, as per the above theoretical explanation, the parallel stub will act as an efficient sensor. The design parameters of the designed sensor stubs gap *g* and stubs tips length *d* has influence on the sensitivity of the designed sensor.

The sensitivity of the sensor depends upon the variation in dielectric permittivity with respect to resonant frequency, transmission coefficient, phase and quality factor of the designed sensor [64]. The sensitivity of a frequency-dependent dielectric sensor can be defined as follows [65]:

(6) $$S=\frac{\Delta f}{\Delta \varepsilon_{r}}$$

where Δf is the frequency shift and is given by $\Delta f=f_{r}-f_{0}$, in which $f_{r}$ is the resonant frequency of the sensor without loading any samples, and $f_{0}$ is the resonant frequency when sample is loaded onto the sensor. $\Delta \varepsilon_{r}$ is the dielectric permittivity and is given by $\Delta \varepsilon_{r}=\Delta \varepsilon_{rr}-\Delta \varepsilon_{r0}$, where $\Delta \varepsilon_{rr}$ is the dielectric permittivity of the sensor material with samples loaded, and $\Delta \varepsilon_{r0}$ is the dielectric permittivity without any samples loaded on to the sensor. The sensitivity can be approximated from the corresponding values of the frequency shift and dielectric permittivity variation, as shown in the simulation results presented in Figure 21, based on the analysis performed by changing the dielectric permittivity of the loaded sample onto the sensor.

The same technique can be made applicable in the detection of adulteration in solid and liquid materials depending upon the variation in the dielectric permittivity of the added sample, which will be discussed in the next section.

## 4. Experimental Validation of Parallel Stub (PS) as Microwave Sensor

The experimental validation and analysis using parallel stub resonator band stop filter is explained in this section. Various samples are added onto the parallel stub to validate the sensitivity of the deigned parallel stub sensor. The various parameters such as resonant frequency $f_{r}$, reflection coefficient $S_{11}$, transmission coefficient $S_{21}$, bandwidth BW, wavelength λ, phase and Q factor are computed by loading different samples onto the dielectric sensor.

### 4.1. Experimental Testing and Results Using PS Sensor in Food Samples

Testing of various food samples using the PS sensor is being discussed in this section. The food samples are weighed and prepared with accuracy for the measurement. From the prepared samples, a small quantity (μg or μL) of the sample to be tested is loaded on to the parallel stub region of the sensor.

#### 4.1.1. Senor Testing in Solid Samples

**Figure 22:**
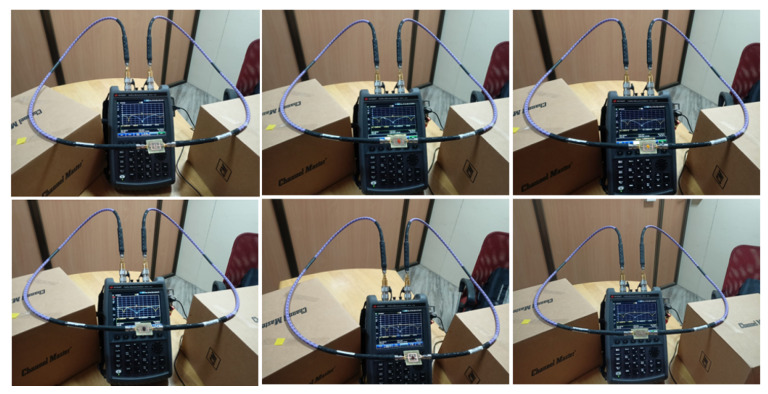
Senor testing in solid samples.

**Figure 23:**
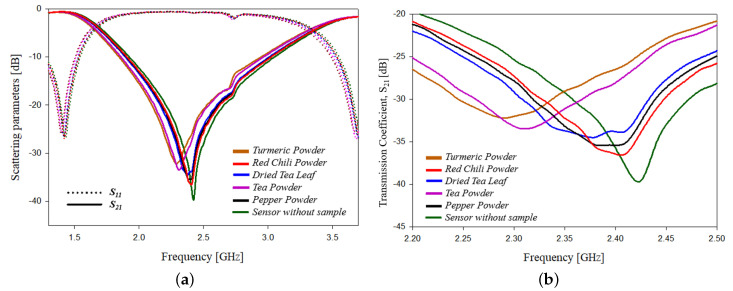
Senor testing in solid samples: (**a**) variation in scattering parameters with frequency; (**b**) variation in transmission coefficient parameters with frequency.

As a part of testing equal quantities of solid samples (spices) such as turmeric powder, red chili powder, dried tea leaf, tea powder and pepper powder are added onto the PS sensor. The test setup for the experimental study for measuring different solids is shown in Figure 22. On adding the samples a variation in scattering parameters, resonant frequency, phase, etc., can be observed. The variation of scattering parameters with frequency is shown graphically in Figure 23. Table 7 shows the detailed values of each of the parameters.

#### 4.1.2. Senor Testing in Liquid Samples

**Figure 24:**
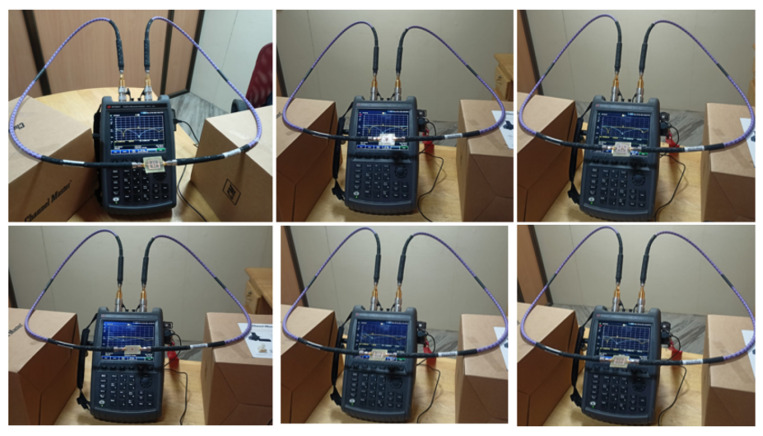
Senor testing in liquid samples.

**Figure 25:**
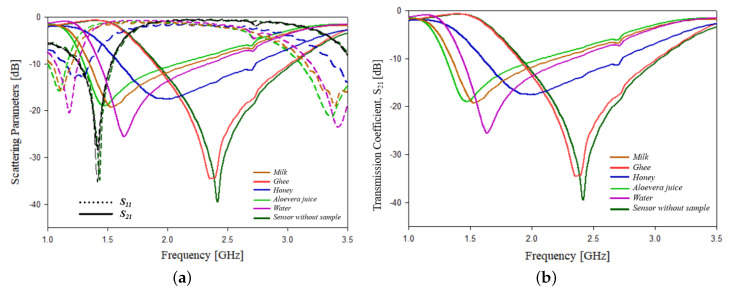
Senor testing in liquid samples: (**a**) variation in scattering parameters with frequency; (**b**) variation in transmission coefficient parameters with frequency.

For testing liquid samples, an equal quantity of liquid samples such as milk, ghee, honey, aloevera juice and water are added onto the PS sensor. The test setup for the experimental study for measuring different liquids is shown in Figure 24. On adding the samples, a variation in scattering parameters, resonant frequency, phase, etc., can be observed, which is shown graphically in Figure 25. The Table 8 shows the detailed values of each of the parameters.

### 4.2. Adulteration Sensing in Food Components

**Figure 26:**
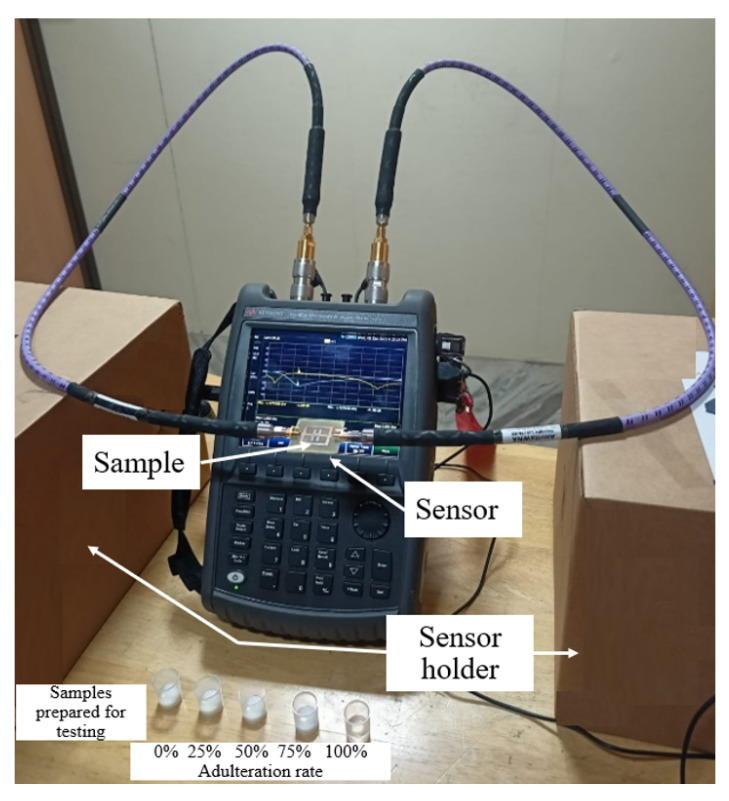
Experimental assessment, measurement setup for adulteration testing.

A proof of concept for the sensing operation of the designed sensor is validated in the laboratory using Amrita-Keysight N9915A Fieldfox Analyzer. The measurement setup is shown in Figure 26.

#### 4.2.1. Adulteration Sensing in Turmeric

**Figure 27:**
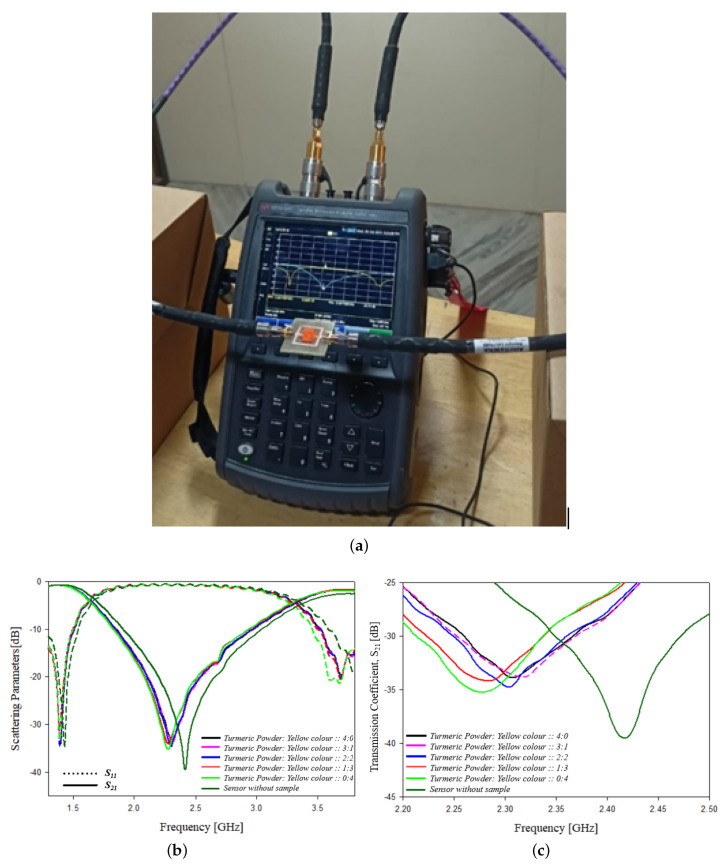
Adulteration sensing in turmeric powder: (**a**) experimental setup, (**b**) variation in scattering parameters with frequency and (**c**) variation in transmission coefficient parameters with frequency.

Adulteration content on turmeric is analyzed experimentally using the designed sensor. Yellow color is added as adulterant to turmeric powder. The turmeric powder and yellow color adulterant are properly mixed and equal quantity of samples with different proportions is tested. Different ratios of turmeric powder and yellow color are chosen as 100% pure turmeric, 75% pure turmeric with 25% adulterant, 50% pure turmeric with 50% adulterant, 25% pure turmeric with 75% adulterant and 100% adulterant are tested, and the frequency shift, scattering parameters, bandwidth, wavelength, phase and Q factor are analyzed and shown in Table 9. The experimental setup and the graphs showing the variation in the scattering parameters with frequency are shown in Figure 27.

#### 4.2.2. Adulteration Sensing in Ghee

**Figure 28:**
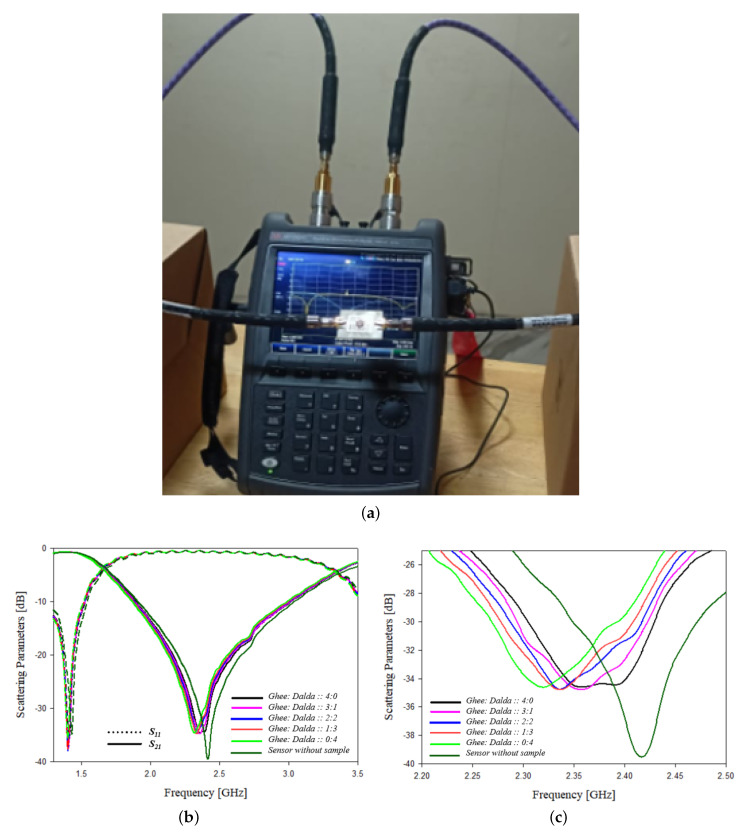
Adulteration testing in ghee (**a**) Experimental setup (**b**) Variation of scattering parameters with frequency (**c**) Variation of transmission coefficient parameters with frequency.

Adulteration content in ghee is analyzed experimentally using the designed sensor. Vegetable oil is added as an adulterant to ghee. The ghee and vegetable oil are properly mixed in different proportions and equal quantities of the prepared samples are tested. Different ratios of ghee and vegetable oil are chosen as 100% pure ghee, 75% pure ghee with 25% adulterant, 50% pure ghee with 50% adulterant, 25% pure ghee with 75% adulterant and 100% adulterant are tested. The frequency shift, scattering parameters, bandwidth, wavelength, phase and Q factor are analyzed by loading specific quantities of these samples on to the sensor, and the variations in each of the parameters are shown in Table 10. The experimental setup and the graphs showing the variation of scattering parameters with frequency are represented in Figure 28.

#### 4.2.3. Adulteration Sensing in Honey

**Figure 29:**
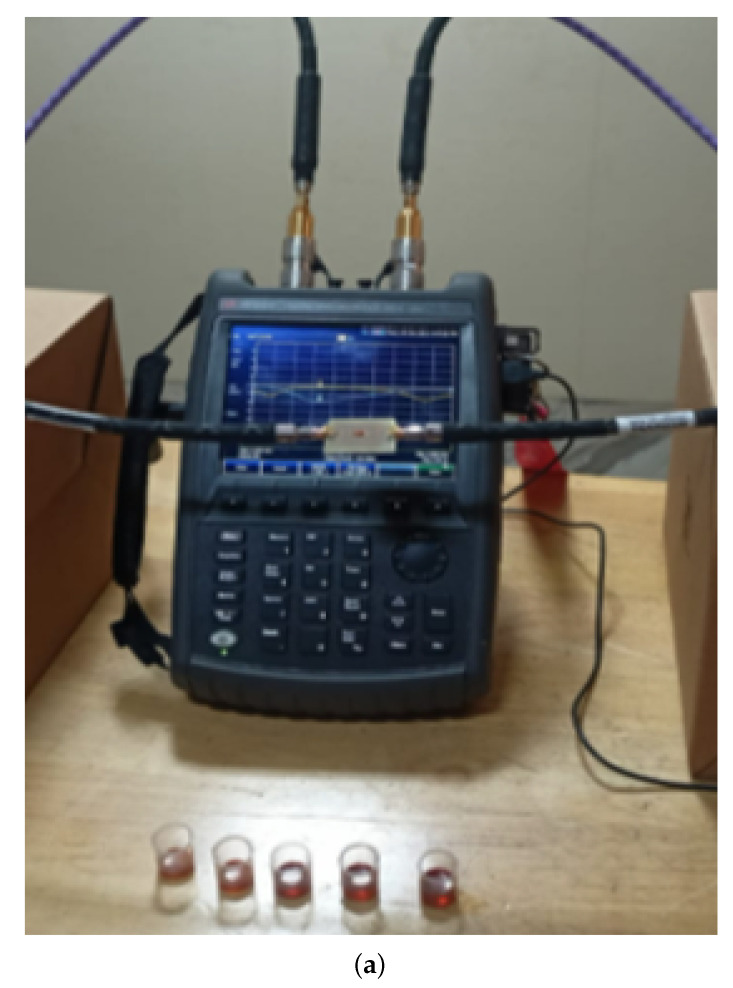

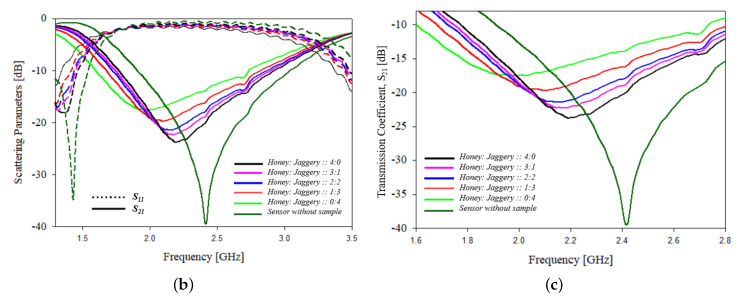
Adulteration testing in honey: (**a**) experimental setup, (**b**) variation in the scattering parameters with frequency and (**c**) variation in transmission coefficient parameters with frequency.

Adulteration content in honey is analyzed experimentally using the designed sensor. Jaggery with same consistency as that of honey is added as an adulterant to honey. The honey and jaggery adulterant are properly mixed and equal quantities of samples with different proportions are measured. Different ratios of honey and jaggery are mixed as 100% pure honey, 75% pure honey with 25% adulterant, 50% pure honey with 50 % adulterant, 25% pure honey with 75% adulterant and 100% adulterant are measured, and the frequency shift, scattering parameters, bandwidth, wavelength, phase and Q factor are analyzed and shown in Table 11. The experimental setup and the graphs showing the variation of scattering parameters with frequency are shown in Figure 29.

#### 4.2.4. Adulteration Sensing in Milk

**Figure 30:**
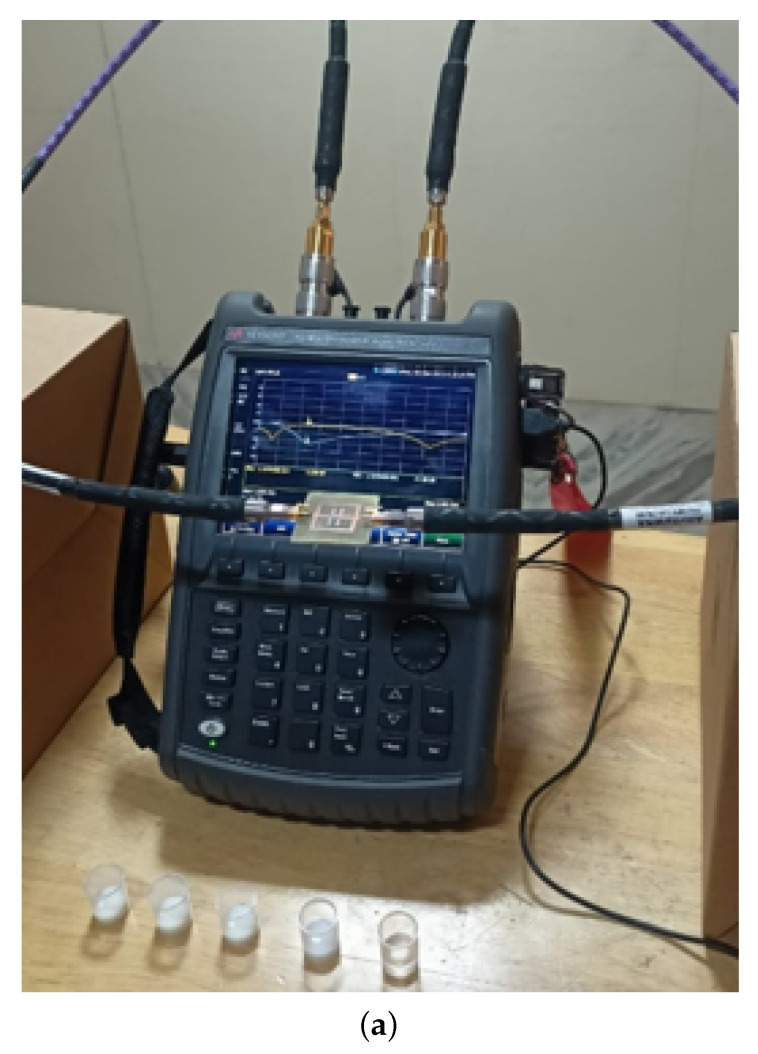

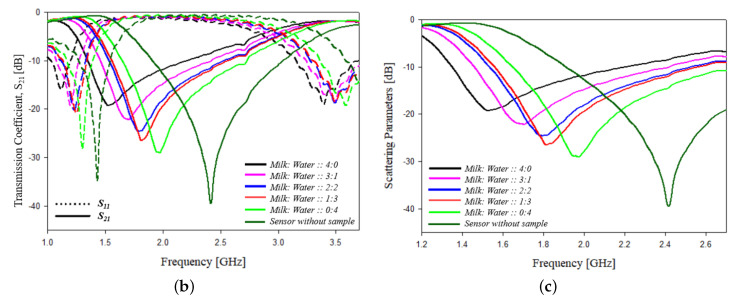
Adulteration testing in milk: (**a**) experimental setup, (**b**) variation in scattering parameters with frequency and (**c**) variation in transmission coefficient parameters with frequency.

Adulteration content on milk is analyzed experimentally using the designed sensor. Water is added as adulterant to milk. Milk and water are properly mixed and an equal quantity of samples with different proportions is measured. Different ratios of milk and water are mixed as 100% pure milk, 75% pure milk with 25% adulterant, 50% pure milk with 50% adulterant, 25% pure milk with 75% adulterant and 100% adulterant, and the frequency shift, scattering parameters, bandwidth, wavelength, phase and Q factor are analyzed and shown in Table 12. The experimental setup and the graphs showing the variation in the scattering parameters with frequency are shown in Figure 30.

#### 4.2.5. Adulteration Sensing in Sesame Oil

**Figure 31:**
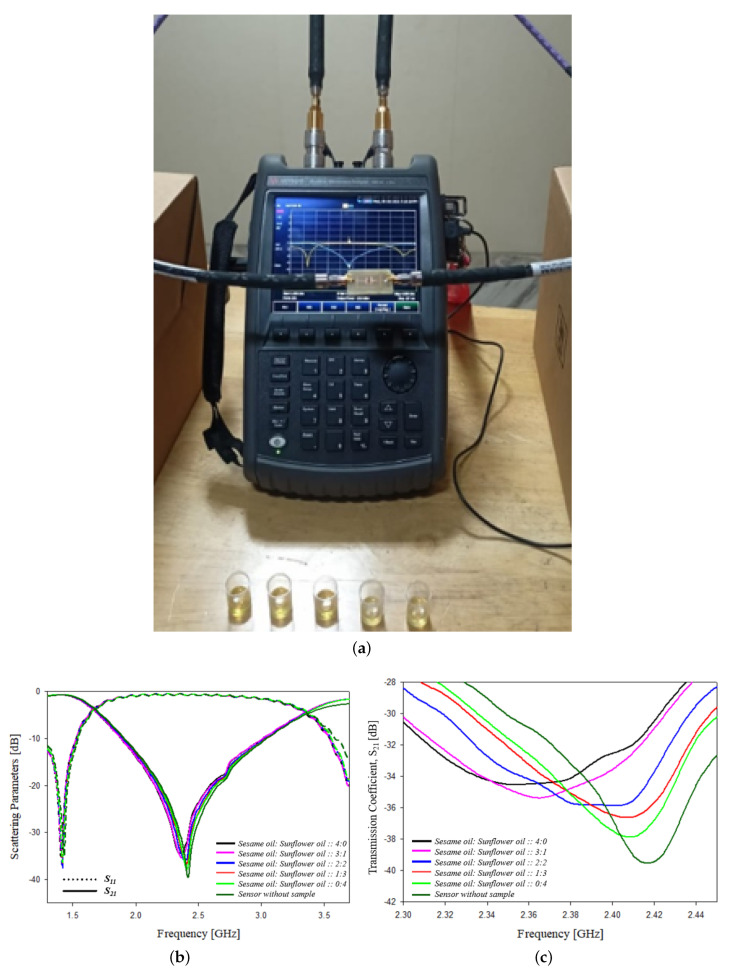
Adulteration testing in sesame oil: (**a**) experimental setup, (**b**) variation of scattering parameters with frequency and (**c**) variation of transmission coefficient parameters with frequency.

Adulteration content on sesame oil is analyzed experimentally using the designed sensor. Sunflower oil is added as an adulterant to milk. Sesame oil and sunflower oil are properly mixed, and an equal quantity of samples with different proportions is measured. Different ratios of sesame oil and sunflower oil are mixed as 100% pure sesame oil, 75% pure sesame oil with 25% adulterant, 50% pure sesame oil with 50% adulterant, 25% pure sesame oil with 75% adulterant and 100% adulterant, are measured and the frequency shift, scattering parameters, bandwidth, wavelength, phase and Q factor are analyzed and shown in Table 13. The experimental setup and the graphs showing the variation of scattering parameters with frequency is shown in Figure 31.

### 4.3. Analysis of Experimental Results and Comparisons

From the experimental testing, it can be observed that on adding a specific amount of different samples on to the parallel stubs of the sensor, the parameters such as resonant frequency, scattering parameters, phase, Q factor, etc., changes adversely. This change indicates the high sensitivity of the designed sensor. The variation with respect to each of the components is discussed below.

**Figure 32:**
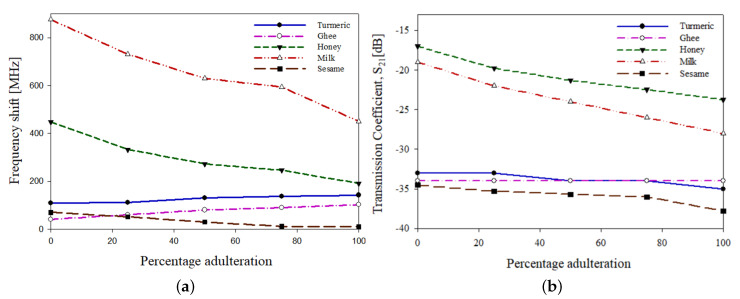
Adulteration testing in sesame oil: (**a**) variation in scattering parameters with frequency (**b**) and variation in the transmission coefficient parameters with frequency.

The variation in resonant frequency with percentage of adulteration on to the samples is shown in Figure 32a. The resonant frequency of a system can be defined as the frequency of oscillation at its natural resonance and transmission coefficient gives the amount of electromagnetic waves passed. On loading the samples onto the sensor, the resonant frequency is being shifted, and it can be observed that the frequency is shifted to the lower range. Similarly, a variation can be observed in the transmission coefficient. The variation in transmission coefficient with percentage of adulteration on to the samples is shown in Figure 32b. The shift in the resonant frequency and scattering parameters is due to the variation in the change in dielectric permittivity of the loaded material onto the sensor.

**Figure 33:**
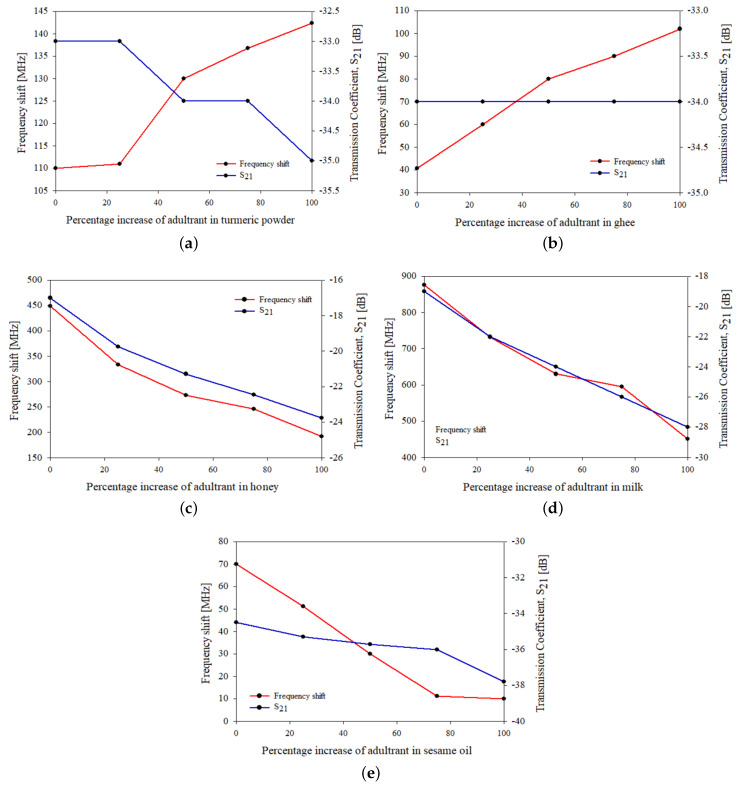
Variation in resonant frequency and transmission coefficient on adding adulterant to (**a**) turmeric powder, (**b**) ghee, (**c**) honey, (**d**) milk and (**e**) sesame oil.

The variation in resonant frequency shift and transmission coefficient with a percentage increase in the adulterants on to the samples is shown in Figure 33. From the graphs, turmeric powder and sesame oil had shown an inverse relation, while ghee and milk had shown a direct relation between the resonant frequency shift and the transmission coefficient. On the other side, no dependence between the resonant frequency and the transmission coefficient is observed in the case of ghee as the transmission coefficient had remained constant for all the cases of percentage increases in adulterants.

A similar variation can be observed in the phase and quality factor (Q) values when analyzing the measured results. All these variations indicate the sensitivity of the designed sensor. It is giving the variation in the sensor output with variations in parameters such as resonant frequency, scattering parameters, phase and Q factor by loading the samples on to the sensor. The Q factor depends upon the transmission coefficient and the variation in $S_{21}$ is interpreted to calculate the value of Q.

The sensitivity of the designed sensor varies depending upon the samples that is loaded onto the sensor, as the sensitivity depends upon the frequency shift and dielectric permittivity variation of the loaded samples. It is validated for all the use cases mathematically with the simulation results. During experimental testing, aluminum foil of known dielectric permittivity ($\epsilon_{r}$ = 10.8) is loaded onto the sensor, the dielectric shift is calculated and a corresponding frequency shift of 0.790 GHz is observed. With this as reference, dielectric permittivity of any loaded sample can be calculated by the principle of frequency shift. This serves as a proof of concept of the experimental results and the simulated graph in Figure 21. The values of dielectric shift in Table 14 was calculated from the simulation graph in Figure 21 corresponding to the frequency shift observed when loading different samples. The sensitivity and normalized sensitivity [48] of the loaded samples is presented in Table 14. Sensitivity is computed as per Equation (6) and normalized sensitivity is computed as per Equation (7), where $S_{N}$ is the normalized sensitivity, Δf is the frequency shift, $f_{r}$ is the resonant frequency and $\Delta \varepsilon_{r}$ is the shift in dielectric permittivity.

(7) $$S_{N}=\left(\frac{\Delta f}{f_{r}\Delta \varepsilon_{r}}\right)\times 100\left[\%\right]$$

The theoretical and experimental validations of the above mentioned sensor are carried out in S band. To understand the sensor performance in other frequency bands, the PS sensor is analyzed in L and C band using ANSYS HFSS. The analysis carried out and the observed results are presented in Table 15. From the frequency shift obtained, the corresponding sensitivity values can be interpreted. From the table, it can be inferred that with increase in the unloaded resonant frequency, the frequency shift increases and thus the sensitivity of the sensor increases.

A comparison of the designed sensor with the various other types of sensors is presented in Table 16. All these serve as proofs of concept indicating the sensitivity of the designed PS sensor.

## 5. Conclusions

The work discussed in the paper demonstrates the design and miniaturisation of planar microstrip resonator filter to make it operational as a sensor with high sensitivity in testing adulteration in food samples. The designs of the compact microstrip filters with band-stop and band pass behaviors have been designed, fabricated and numerically and experimentally assessed. In particular, six different filter designs were considered. An optimised size reductions of 69% for BSF and 79% for BPF filters were achieved, in comparison with the standard design, using open and short-circuited, quarter wavelength stubs and other state of the art sensors. The obtained results were quite good, in particular, the furnished design equations can predict resonant frequencies of the devices, with less than a 3% error on Rogers-TM-based substrates and an error less than 6% for other lossy dielectric materials. The optimised filter which operates as a dielectric sensor is experimentally tested by loading different solid and liquid samples. A detailed analysis of the sensor performance based on frequency shift, dielectric shift, Q factor, sensitivity and normalized sensitivity is carried out. The sensor is also operational as an efficient food adulteration detection sensor as it shows adequate sensitivity on loading different food samples. Detailed analysis in both solid and liquid food samples was carried out with the efficient detection of adulteration in both cases. The performance of the sensor is not found to deteriorate while reusing the same for different sample testing.

## Figures and Tables

**Table 1:** Prototypes dimensions comparisons; $\varepsilon_{r}=4.0$, h=1.6 mm.

SL. No.	Type	*L* [mm]	*g* [mm]	*d* [mm]
1	Perpendicular F1	40.00	4.16	2.00
2	Parallel F2	37.80	4.00	2.00
3	Perpendicular stub F3	37.80	8.00	7.00
4	Parallel stub F4	21.00	2.00	12.50
5	Perpendicular stub loop F5	28.00	8.00	23.90
6	Parallel stub loop F6	22.10	2.00	18.10

**Table 2:** Band stop filters comparisons; $\varepsilon_{r}=4.0$, h=1.6 mm.

SL. No.	Type	$S_{11}$ [dB]	$S_{21}$ [dB]	BW [GHz]	L [mm]	Area [mm${}^{2}$]
1	LC stub circuit	−0.20	−40.0	1.000	NA	NA
2	Open/short stub	−0.82	−70.0	2.000	NA	90.7×45.1
3	Parallel F1	−0.25	−44.0	1.092	40.0×40.0	54.0×54.0
4	Perpendicular F2	−0.45	−36.0	1.095	40.0×40.0	54.0×54.0
5	Perpendicular stub F3	−0.46	−36.0	1.185	37.8×37.8	51.8×51.8
6	Parallel stub F4	−0.24	−37.0	1.636	21.0×21.0	35.0×35.0

**Table 3:** Band pass filters comparisons; $\varepsilon_{r}=4.0$, h=1.6 mm.

SL. No.	Type	$S_{11}$ [dB]	$S_{21}$ [dB]	BW [GHz]	L [mm]	Area [mm${}^{2}$]
1	LC stub circuit	−0.2	−40.0	1.0	NA	NA
2	Open/short stub	−1.3	−12.0	1.0	NA	90.75×60.25
3	Perpendicular stub loop	−24.0	−0.7	1.095	27.9×27.9	41.9×41.9
4	Parallel stub loop	−25.0	−0.55	1.394	22.1×22.1	36.1×36.1

**Table 4:** Comparison of referred band stop and band pass filter.

SL. No.	Filter Type	Performance	Substrate Characteristics	$f_{c}$ [GHz]	Bandwidth [GHz]	Area [mm${}^{2}$]
1	Ref [19]	BSF	$\varepsilon_{r}=3.48$, h = 1.524 mm	2.00	1.0	NA
2	Ref [24]	BPF	$\varepsilon_{r}=2.65$, h = 1.0 mm	2.45	NA	112 × 41
3	Ref [25]	BPF	$\varepsilon_{r}=4.4$, h = 1.56 mm	2.00	0.4	166 × 50
4	Ref [26]	BPF	$\varepsilon_{r}=10.2$, h = 1.27 mm	2.23	1.38	8.4 × 24.1
5	Ref [27]	BPF	$\varepsilon_{r}=4.4$, h = 1.60 mm	2.45	0.5	NA
6	Filter 4	BSF	$\varepsilon_{r}=4.0$, h = 1.60 mm	2.48	1.5	35.0 × 35.0
7	Filter 6	BPF	$\varepsilon_{r}=4.0$, h = 1.60 mm	2.48	1.5	36.1 × 36.1

**Table 5:** Optimized filter performances: $\varepsilon_{r}=4.0$, h = 1.6 mm.

SL. No.	Parameter	BSF—Prototype 4 (PS)	BPF—Prototype 6 (PSL)
1	Simulation freq., $f_{sim}$ [GHz]	2.48	2.48
2	Measured freq., $f_{meas}$ [GHz]	2.42	2.44
3	Calculated freq., $f_{cal}$ [GHz]	2.34	2.37
4	Area [mm${}^{2}$]	1225.0	1303.0
5	Design equation	$f=\frac{\gamma_{2}CgW\sqrt{\varepsilon_{e}}}{2LW_{t}d}$	$f=\frac{\gamma_{3}C}{2gLW_{t}\sqrt{\varepsilon_{e}}}$

**Table 6:** Optimized filter performances; $\varepsilon_{r}=4.0$, h=1.6 mm.

$\varepsilon_{\mathrm{rs}}$	PS—Freq. [GHz]	PSL—Freq. [GHz]
1	2.4636	2.4848
5	2.1485	2.4545
10	1.9121	2.3939
15	1.7152	2.3330
20	1.5970	2.3030
25	1.4788	2.2424
30	1.4000	2.2121
35	1.3212	2.1515
40	1.2818	2.1212
45	1.2424	2.0606
50	1.1636	2.1212
55	1.1242	2.0000
60	1.0848	1.9677
70	1.0455	1.8788
80	0.9667	1.8182
90	0.9273	1.7576
100	0.8879	1.7273

**Table 7:** Solid sample testing.

Sample	fr [GHz]	$S_{11}$ [dB]	$S_{21}$ [dB]	BW [GHz]	λ [m]	Phase [deg]	Q Factor
Turmeric Powder	2.31	−0.3	−33.00	1.6620	0.1298	48.3560	44.67
Red Chili Powder	2.40	−0.4	−36.36	1.6781	0.1249	50.2672	65.77
Dried Tea Leaves	2.37	−0.4	−34.00	1.6750	0.1263	49.7208	50.12
Tea Powder	2.39	−0.4	−34.00	1.6711	0.1255	50.0097	50.12
Pepper Powder	2.38	−0.4	−34.00	1.6786	0.1255	50.0055	50.12
Sensor without Sample	2.42	−0.3	−39.60	1.6855	0.1239	50.6586	95.50

**Table 8:** Liquid sample testing.

Sample	fr [GHz]	$S_{11}$ [dB]	$S_{21}$ [dB]	BW [GHz]	λ [m]	Phase [deg]	Q Factor
Milk	1.54	−1.0	−19.0	1.5860	0.1942	32.3252	8.91
Ghee	2.38	−0.5	−34.0	1.6962	0.1260	49.8045	50.12
Honey	1.97	−1.4	−17.0	1.7032	0.1521	41.2679	7.08
Aloevera Juice	1.47	−1.3	−19.0	1.5415	0.2034	30.8662	8.91
Water	1.97	−1.0	−28.0	1.4650	0.1523	41.2177	25.12
Sensor without Sample	2.42	−0.3	−39.6	1.6855	0.1239	50.6586	95.50

**Table 9:** Adulteration testing in turmeric.

Turmeric: Yellow Colour Ratio	fr [GHz]	$S_{11}$ [dB]	$S_{21}$ [dB]	BW [GHz]	λ [m]	Phase [deg]	Q Factor
100%:0% (4:0)	2.310	−0.33	−33	1.662	0.1298	48.3560	44.67
75%:25% (3:1)	2.309	−0.33	−33	1.662	0.1299	48.3350	44.67
50%:50% (2:2)	2.290	−0.33	−34	1.662	0.1310	47.9373	50.12
25%:75% (1:3)	2.283	−0.33	−34	1.662	0.1313	47.7949	50.12
0%:100% (0:4)	2.277	−0.33	−35	1.662	0.1317	47.6777	56.23

**Table 10:** Adulteration testing in ghee.

Ghee:Dalda Ratio	fr [GHz]	$S_{11}$ [dB]	$S_{21}$ [dB]	BW [GHz]	λ [m]	Phase [deg]	Q Factor
100%:0% (4:0)	2.379	−0.5	−34	1.6962	0.1260	49.8045	50.12
75%:25% (3:1)	2.360	−0.5	−34	1.6962	0.1271	49.4026	50.12
50%:50% (2:2)	2.340	−0.5	−34	1.6962	0.1282	48.9840	50.12
25%:75% (1:3)	2.330	−0.5	−34	1.6962	0.1287	48.7746	50.12
0%:100% (0:4)	2.318	−0.5	−34	1.6962	0.1294	48.5234	50.12

**Table 11:** Adulteration testing in honey.

Honey:Jaggery Ratio	fr [GHz]	$S_{11}$ [dB]	$S_{21}$ [dB]	BW [GHz]	λ [m]	Phase [deg]	Q Factor
100%:0% (4:0)	1.971	−1.4	−17.00	1.7032	0.1521	41.2679	7.08
75%:25% (3:1)	2.087	−1.4	−19.76	1.7135	0.1437	43.6878	9.73
50%:50% (2:2)	2.147	−1.4	−21.28	1.7100	0.1397	44.9522	11.60
25%:75% (1:3)	2.174	−1.4	−22.47	1.6855	0.1379	45.5174	13.29
0%:100% (0:4)	2.228	−1.4	−23.76	1.6881	0.1346	46.6503	15.41

**Table 12:** Adulteration testing in milk.

Milk:Water Ratio	fr [GHz]	$S_{11}$ [dB]	$S_{21}$ [dB]	BW [GHz]	λ [m]	Phase [deg]	Q Factor
100%:0% (4:0)	1.544	−1	−19	1.586	0.1942	32.3252	8.91
75%:25% (3:1)	1.688	−1	−22	1.550	0.1777	35.3396	12.59
50%:50% (2:2)	1.790	−1	−24	1.510	0.1675	37.4706	15.85
25%:75% (1:3)	1.826	−1	−26	1.510	0.1643	38.2158	19.95
0%:100% (0:4)	1.969	−1	−28	1.465	0.1523	41.2177	25.11

**Table 13:** Adulteration testing in sesame oil.

Sesame:Sunflower Ratio	fr [GHz]	$S_{11}$ [dB]	$S_{21}$ [dB]	BW [GHz]	λ [m]	Phase [deg]	Q Factor
100%:0% (4:0)	2.350	0.5	−34.5	1.6863	0.1276	49.1933	53.80
75%:25% (3:1)	2.368	0.5	−35.3	1.6863	0.1266	49.5868	58.21
50%:50% (2:2)	2.390	0.5	−35.7	1.6863	0.1255	50.0306	60.96
25%:75% (1:3)	2.408	0.5	−36.0	1.6863	0.1245	50.4242	63.10
0%:100% (0:4)	2.410	0.5	−37.8	1.6863	0.1244	50.4493	77.62

**Table 14:** Sensitivity chart on loading various samples.

Sample Tested [μg]	Frequency Shift, [GHz]	Dielectric Shift	Sensitivity [MHz]	Normalised Sensitivity [%]
Aluminium foil	0.790	6.80	116	4.8
Turmeric powder	0.116	1.42	82	3.4
Tea dried leaf	0.030	2.66	11	0.47
Tea leaf powder	0.099	1.50	66	2.7
Pepper	0.018	2.66	6.8	0.3
Red chilli	0.012	2.92	4	0.2
Ghee	0.040	2.29	17.5	0.7
Vegetable ghee	0.170	0.42	409	17
Honey	0.436	2.37	184	7.6
Milk	0.880	17.15	51	2.1
Water	0.769	12.33	62	2.6
Aloevera	0.950	20.17	47	2
Coconut oil	0.100	1.79	56	2.3
Palm oil	0.090	1.95	46	2
Sesame oil	0.110	1.00	110	4.6
Olive oil	0.090	1.21	74	3.1

**Table 15:** Frequency shift in various frequency bands on varying the dielectric permittivity of samples loaded on to the sensor.

Unloaded Resonant Frequency [GHz]	Frequency Shift [GHz] for Sample $\epsilon_{r}$ = 5	Frequency Shift [GHz] for Sample $\epsilon_{r}$ = 10	Frequency Shift [GHz] for Sample $\epsilon_{r}$ = 15
1.8 (L band)	0.103	0.1939	0.2848
2.4 (S band)	0.2515	0.4879	0.6848
5.2 (C band)	0.6242	0.9273	1.2303

**Table 16:** Performance comparison based on sensitivity of the sensor described in this work with other high sensitive sensors available in the literature.

Reference Number	Type of Sensor	Unloaded Resonance Frequency [GHz]	Sensitivity [MHz]	Tested Sample	Sample Size/ Quantity	Relative Sensitivity per μg/μL [48]
[65]	Rotated ELC	3.9	136	Flours	μg	0.05
[66]	CSRR	1.164	69	Solid	-	0.86
[67]	SIW antenna	4.69	4.6	Ethanol	nL	0.17
[68]	Waveguide cavity	2.4	-	Edible Fluids	3 mm	0.08
[69]	SRR	1.84	56	Oils	-	-
[70]	SRR	0.87	0.79	Ethanol	5 × 5 mm${}^{2}$	-
[71]	SIW cavity	1.5134	8	Solid	8 × 8 mm${}^{2}$	-
[72]	LC	0.016	-	Olive oil	11 ml	-
[73]	IDC-SRR	2.45	2.5	Solid	5 × 5 mm${}^{2}$	
[74]	Bragg grating	-	32.25 nm/RIU	Diesel	-	-
[74]	Bragg grating	-	54.98 nm/RIU	Petrol	-	-
[75]	optical fiber	-	1.1107 dBm	Olive oil		-
This work	ELC	2.42	116	Aluminium foil	50 μg	0.66
This work	ELC	2.42	82	Turmeric powder	50 μg	0.09
This work	ELC	2.42	11	Tea dried leaf	50 μg	0.02
This work	ELC	2.42	66	Tea leaf powder	50 μg	0.02
This work	ELC	2.42	6.8	Pepper	50 μg	0.08
This work	ELC	2.42	4	Red chilli	50 μg	0.01
This work	ELC	2.42	17.5	Ghee	50 μL	0.03
This work	ELC	2.42	184	Honey	50 μL	0.36
This work	ELC	2.42	51	Milk	50 μL	0.76
This work	ELC	2.42	62	Water	50 μL	0.64
This work	ELC	2.42	47	Aloevera	50 μL	0.79
This work	ELC	2.42	56	Coconut oil	50 μL	0.08
This work	ELC	2.42	409	Vegetable ghee	50 μL	0.14
This work	ELC	2.42	46	Palm oil	50 μL	0.07
This work	ELC	2.42	110	Sesame oil	50 μL	0.09
This work	ELC	2.42	74	Olive oil	50 μL	0.07

## Data Availability

Not applicable.
